# Application of Laparoscopic Gastric Jejunum Uncut Roux-en-Y Anastomosis

**DOI:** 10.1155/2022/9496271

**Published:** 2022-04-01

**Authors:** Chao Yu, Tian Yang, Qiang Yan, Deguan Li, Yigao Wang, Xiaodong Yang, Shangxin Zhang, Yonghong Zhang, Zhen Zhang

**Affiliations:** ^1^Department of General Surgery, Taizhou People's Hospital, Taizhou Medical College of Nanjing Medical University, Taizhou, China; ^2^Anhui Medical University First Affiliated Hospital of General Surgery, 055162923887, 218 Jixi Road, Shushan District, Hefei City, Anhui Province, China

## Abstract

**Background:**

Uncut Roux-en-Y gastrojejunostomy, recently developed in China, is useful in the treatment of distal gastric cancer. This study is aimed at comparing laparoscopic gastric jejunum uncut Roux-en-Y anastomosis with conventional anastomosis in the surgical treatment of distal gastric malignancy.

**Methods:**

In this retrospective study, the clinical data of 178 patients and their follow-up records were analyzed. 112 cases (uncut group) were the observation group for stomach jejunum uncut Roux-en-Y anastomosis, the control group for the stomach, 66 cases (conventional group) were for jejunum Roux-en-Y anastomosis and Billroth I and Billroth II anastomosis. A comparison between the two groups was conducted based on the general situation of the patients, TNM stage, and one-year survival rate.

**Results:**

There was no significant difference reported between the two groups in terms of the general situation and TNM stage. A comparison on postoperative complications between the two groups revealed that the postoperative bleeding was 0.9% and 6.1%, the bile reflux gastritis was 1.8% and 9.1%, the anastomotic leakage was 0.0% and 3.0%, the delayed gastric emptying was 0.9% and 7.6%, and the overall complications was at 3.6% and 25.8%, which was significantly lower in the observation group than in the control group, and the difference was statistically significant. Notably, there was no significant difference in 1-year survival rate between the two groups.

**Conclusion:**

Laparoscopic gastric jejunal uncut Roux-en-Y anastomosis significantly reduces the risk of postoperative complications of the digestive tract. Its operation is easy and exhibits an effective curative effect.

## 1. Introduction

Gastric cancer is one of the major gastrointestinal malignancies in China and the second leading cause of malignancy-related fatalities globally [1, 2]. The surge in the incidence and mortality rates of gastric cancer in China is attributed to the aging population, and the sudden switch to unhealthy living habits and lifestyles [3, 4]. Reports suggest that surgery is the main treatment for gastric cancer, specifically, laparoscopic surgery is currently an effective treatment [5]. In recent years, with the emergence of novel surgical methods and instruments, laparoscopic surgery for gastric cancer developed rapidly [6, 7]. In clinical practice, gastric jejunum Roux-en-Y anastomosis is one of the most commonly used surgical methods in the treatment of distal gastric malignancies due to its simple operation. However, due to the long operation time and the influence on the normal anatomical structure of the gastrointestinal tract, it has adverse effects on the postoperative antireflux of patients and severely regulates the quality of life and survival time of patients [8, 9]. Gastric jejunum uncut Roux-en-Y anastomosis has gradually evolved into being the primary surgical treatment for clinical distal gastric malignancy through decades of continuous development since it was first reported in 1988 [10, 11]. Being a new surgical method, gastric jejunum uncut Roux-en-Y anastomosis has a simple operation, short operation time, and no jejunum transection during the operation, thereby effectively promoting postoperative recovery of patients and reducing the occurrence of delayed gastric emptying [12, 13]. Herein, we compared the clinical effects of different surgical methods geared towards exploring their impacts and clinical application prospects.

## 2. Materials and Methods

### 2.1. General Information

Between May 2014 and December 2018, 178 cases diagnosed with distal gastric malignant tumors were surgically treated at the first affiliated hospital of Anhui Medical University during the period of cavity mirrors. The clinical data of patients were retrospectively analyzed and follow-up, according to the operation, they were divided into the stomach jejunum uncut Roux en-Y anastomosis group (observation group) with 112 cases, among which 78 cases were males, female 34 cases were females; age: 28-76 (63.4 ± 6.7) years; TNM staging of tumor: phase I of 17 cases, II period 26 cases, III 69 cases. Regular gastrojejunostomy (stomach and jejunum Roux en-Y anastomosis, Billroth I, and Billroth II) group (control group) 66 cases, 35 cases of male, female 31 cases; age: 32-78 (64.5 ± 7.2) years; tumor TNM stages: stage I in two cases, II period 24 cases, 40 cases III period. All the patients who participated in this study obtained informed consent and underwent the first operation. Before surgery, they were diagnosed with gastric antrum carcinoma through electronic gastroscopy and biopsy. CT and chest radiographs were performed to evaluate the involvement of local and adjacent tissues as well as organs. This study is a retrospective study based on clinical diagnosis and treatment, and the ethical issues involved have been approved by the Ethics Committee of the First Affiliated Hospital of Anhui Medical University.

### 2.2. Operation Method

**Figure 1:**
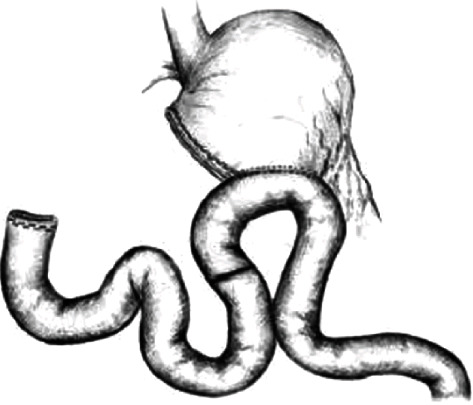
Anastomosis of gastric jejunum uncut Roux-en-Y.

According to the conventional laparoscopic distal gastric malignant tumor, the jejunum Roux-en-Y anastomosis severed the distal stomach, then lift about 25 cm away from closely ligament in jejunum to enter loops on the greater curvature side of the side with the residual stomach anastomosis, again at the anastomotic below about 10 cm, jejunum Braun anastomosis, while the transection jejunum, and about 2 cm below the stomach, jejunum anastomosis of jejunum input loops with no. seven silk ligation, blocking jejunum, form the stomach jejunum uncut Roux-en-Y anastomosis (Figure 1).

### 2.3. Observation Indices

They included postoperative bleeding, bile reflux gastritis, anastomotic leakage, delayed gastric emptying, and other complications of the two groups of patients as well as 1-year survival rate, and recanalization of the ligation site of the digestive tract.

### 2.4. Statistical Analysis

This study used SPSS25.0 statistical software for statistical analysis. The measurement data were presented by *t*-test (^−^*x* ± *s*), and the counting data were presented by *χ*^2^ test (%). A *P* value of less than 0.05 was considered statistically significant.

## 3. Results

### 3.1. Comparing the General Information of Patients

Results showed no significant difference in age, gender other general conditions, and postoperative TNM stage of tumor between the two groups with *P* > 0.05 showing no statistical significance (Table 1).

### 3.2. Comparison of Postoperative Complications

Patients in the two groups were followed up for one year to assess the occurrence of complications. It was found that the occurrence of complications in the two groups was significantly different, moreover, the occurrence of overall complications was significantly different with *P* < 0.05, which was statistically significant (Table 2).

### 3.3. Comparison of One-Year Survival Rate after Surgery and Valuation of Gastrointestinal Patency

**Figure 2:**
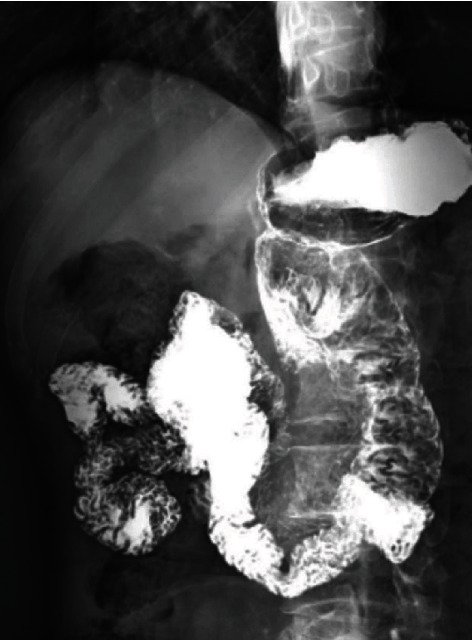
The gastrointestinal barium test showed that the barium agent passed the anastomotic site smoothly, and there was no retuning at the ligation site of line 7.

Further, the patients were followed up for one year after surgery for gastroscopy or upper gastrointestinal angiography to observe the recurrence and survival status of the ligation site of the gastrointestinal tract (Figure 2). There was no significant difference in one-year survival, perhaps attributed to a short follow-up time.

## 4. Discussion

Numerous surgical methods exist for the treatment of distal gastric malignancy, and many of which have their pros and cons [14, 15]. In 2014, for the first time, laparoscopic gastrojejunal uncut Roux-en-Y anastomosis was performed at the department of endoscopic surgery of the first affiliated hospital of Anhui Medical University. Results found that in comparison with conventional gastrojejunal anastomosis, laparoscopic gastrojejunal uncut Roux-en-Y anastomosis significantly reduced the risk of postoperative complications. In Billroth I type and II type gastric jejunum anastomosis, its operation was simple, reconstruction of digestive physiology characteristic and basic preserved, but Billroth I type increased gastric jejunum anastomotic tension, causing an elevated risk of anastomotic leakage. Despite reducing the tension of type II anastomotic site, there was an increase in the risk of anastomotic leakage, bile reflux gastritis, and gastric stump cancer [16, 17]. In normal gastrojejunal Roux en-Y anastomosis for Billroth I and Billroth II anastomosis, the advantages and disadvantages of decreased anastomotic tension, reduce the adverse reactions caused by digestive fluid diversion, but the happening of the delayed gastric emptying elevated risk, conventional stomach jejunum Roux en-Y match the jejunum transection, jejunum under impulse transfer obstacle, leading to delayed gastric emptying [18, 19]. The digestive tract reconstructed by laparoscopic gastric jejunum uncut Roux-en-Y anastomosis reduced the traction effect of abdominal organs on the anastomotic site. It also showed no effect on the complex wave conduction of intestinal movement and alleviated the distension pain, nausea, vomiting, and other adverse symptoms in the upper abdomen, thereby improving the quality of life in tumor patients with satisfactory clinical effect [20, 21]. Laparoscopic gastric jejunum uncut Roux-en-Y anastomosis protects the integrity of jejunum and continuous peristalsis, reducing bile reflux, however, the risk of complications was not completely resolved. According to the retrospective study on 178 cases with distal gastric malignant tumors, the clinical data show that laparoscopic gastric jejunum uncut Roux en-Y anastomosis is a safe and effective in the treatment of distal gastric malignant tumor. In contrast with normal gastric jejunum Roux en-Y fit and Billroth I compared to Billroth II type, laparoscopic gastric jejunum uncut Roux en-Y anastomosis significantly lower risk of postoperative complications has an efficient therapeutic effect and promising clinical application prospects.

In conclusion, we found that laparoscopic gastric jejunum uncut Roux-en-Y anastomosis has a more effective surgical effect and clinical application prospect in the radical treatment of distal gastric malignant tumors compared to conventional gastric jejunal anastomosis. However, its utilization has not matured, and varying opinions are currently being reported by various investigators. Therefore, the clinical application value of this method needs further elucidation through more research.

We have submitted the manuscript as a preprint in *Research Square* and the following is the citation section.

Uncut Roux en-Y gastrojejunostomy, recently developed in China, is useful in the treatment of distal gastric cancer. This study is aimed at comparing laparoscopic gastric jejunum uncut Roux-en-Y anastomosis with conventional anastomosis in the surgical treatment of distal gastric malignancy. Methods: a total of 178 cases diagnosed with distal gastric malignant tumors were surgically treated in the First affiliated Hospital of Anhui University of cavity mirrors [22].

## Figures and Tables

**Table 1:** The clinical features of two groups of patients.

Group	Uncut group	Conventional group	*T*/*χ*^2^ value	*P* value
The number of cases (*n*)	112	66	—	—
Age/yr (*x* ± *s*)	63.4 ± 6.7	64.5 ± 7.2	-0.33	>0.05
Gender (male/female)	78/34	35/31	2.11	>0.05
TNM stage (I II III)	17 26 69	2 24 40	0.96	>0.05

**Table 2:** Two groups of patients with complications.

Group	Uncut group	Conventional group	*χ* ^2^ value	*P* value
The number of cases (*n*)	112	66	—	—
Postoperative bleeding	1	4	4.06	<0.05
Anastomotic leakage	0	3	5.18	<0.05
Bile reflux gastritis	2	6	5.16	<0.05
Delayed gastric emptying	1	5	5.69	<0.05
Total complication rate	4	`18	21.54	<0.05

## Data Availability

The datasets used and/or analyzed during the current study are available from the corresponding author on reasonable request.
